# Characterization of *Krt19* ^*CreERT*^ allele for targeting the nucleus pulposus cells in the postnatal mouse intervertebral disc

**DOI:** 10.1002/jcp.28952

**Published:** 2019-06-11

**Authors:** Sarthak Mohanty, Robert Pinelli, Chitra Lekha Dahia

**Affiliations:** ^1^ Orthopaedic Soft Tissue Research Hospital for Special Surgery New York New York; ^2^ Department of Cell and Developmental Biology, Weill Cornell Medicine Graduate School of Medical Sciences New York New York

**Keywords:** driver line, genetic, inducible, intervertebral disc, nucleus pulposus

## Abstract

Intervertebral disc degeneration and associated back pain are relatively common but sparsely understood conditions, affecting over 70% of the population during some point of life. Disc degeneration is often associated with a loss of nucleus pulposus (NP) cells. Genetic mouse models offer convenient avenues to understand the cellular and molecular regulation of the disc during its formation, growth, maintenance, and aging. However, due to the lack of inducible driver lines to precisely target NP cells in the postnatal mouse disc, progress in this area of research has been moderate. NP cells are known to express cytokeratin 19 (Krt19), and tamoxifen (Tam)‐inducible *Krt19* ^*CreERT*^ allele is available. The current study describes the characterization of *Krt19* ^*CreERT*^ allele to specifically and efficiently target NP cells in neonatal, skeletally mature, middle‐aged, and aged mice using two independent fluorescent reporter lines. The efficiency of recombination at all ages was validated by immunostaining for KRT19. Results show that following Tam induction, *Krt19* ^*CreERT*^ specifically drives recombination of NP cells in the spine of neonatal and aged mice, while no recombination was detected in the surrounding tissues. Knee joints from skeletally mature Tam‐treated *Krt19* ^*CreERT/+*^; *R26* ^*tdTOM*^ mouse show the absence of recombination in all tissues and cells of the knee joint. Thus, this study provides evidence for the use of *Krt19* ^*CreERT*^ allele for genetic characterization of NP cells at different stages of the mouse life.

## Graphical abstract

An inducible driver line to precisely target nucleus pulposus (NP) cells during the postnatal is required to understand the role of these cells during development and aging. The current study describes the characterization of *Krt19^CreERT^* allele to efficiently target NP cells in neonatal, skeletally mature, middle‐aged, and aged mice using two independent fluorescent reporter lines. Moreover, this allele did not target the surrounding cells in the spine or any cell in the knee joint, validating its specificity to target NP cells in the postnatal mouse.

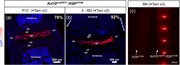

## 1 INTRODUCTION

Lower back pain is considered as one of the top neurological disorders worldwide and accounts for substantial financial loss, mainly due to loss of workdays (Dieleman et al., 2016; HALE Collaborators, 2015; Hartvigsen et al., 2018; Hoy et al., 2012). Back pain is a multifactorial disorder and age, sex, genetics, injury, and lifestyle including smoking play a crucial role in its etiology (Munir, Rade, Maatta, Freidin, & Williams, 2018). Degeneration of the intervertebral disc (IVD) is thought to be a significant contributor to low back pain, although not all degenerated IVDs are symptomatic (Freemont, 2009). Despite being a significant financial burden and with high prevalence, the current treatments for IVD disorders and back pain are primarily palliative, which addresses the symptoms transiently but does not cure the underlying cause. One of the limitations in the development of successful therapeutics is the poor understanding of the cellular and molecular processes that regulate IVD development, growth and maintenance, and how these processes change with aging, and IVD degeneration (reviewed by [Choi, Johnson, & Risbud, 2015; Mohanty & Dahia, 2019; Mwale, 2013; Urban & Roberts, 2003]). Elucidating the cellular and molecular regulation of IVD growth and maintenance will enable the development of therapeutics aimed at regenerating the IVD and treating back pain.

Each IVD has three main components; nucleus pulposus (NP), annulus fibrosus (AF), and cartilaginous endplates (EP). The proteoglycan‐rich NP is located in the center of each IVD and surrounded by orthogonal layers of collagen‐rich AF. A pair of cartilaginous EP sandwich the NP and AF and connect the IVD to the vertebral growth plate (GP). Healthy IVDs produce abundant extracellular matrix molecules that play a vital role in the maintenance of its structure and function (Antoniou et al., 1996; Mwale, 2013). IVDs are the massive avascular structures in the body. The nutrients and systemic factors diffuse into the IVD through the vertebral GP and EP (Urban, Holm, & Maroudas, 1978). However, with aging, the EP undergoes mineralization and further slows the inflow of nutrients and systemic factors into the IVD, particularly in NP area, leading to IVD degeneration (Urban & Winlove, 2007; Wong et al., 2019).

Development of the mouse as a model system has been crucial in elucidating the development and pathophysiology of the IVD (reviewed by Mohanty & Dahia, 2019). The genetic mouse models are not only instrumental in establishing the embryonic origin of the different components of the IVDs (Choi, Cohn, & Harfe, 2008; McCann, Tamplin, Rossant, & Seguin, 2012; Sugimoto, Takimoto, Hiraki, & Shukunami, 2013) but also offer robust approaches for elucidating the role of specific genes and pathways during IVD development, aging or degeneration (Alkhatib, Liu, & Serra, 2018; Alvarez‐Garcia et al., 2018; Bonavita, Vincent, Pinelli, & Dahia, 2018; Choi, Lee, & Harfe, 2012; Sohn, Cox, Chen, & Serra, 2010). NP cells have been the main focus of research related to IVD biology, mainly because they are descendant of the embryonic notochord, a source of developmentally critical signaling ligands, crucial for maintenance of IVD homeostasis, and their loss with age or herniation is one of the causes of IVD degeneration and back pain (reviewed by Mohanty & Dahia, 2019). Hence, to target approaches towards NP cells for degenerative disc disease and back pain, it is crucial to precisely determine the role of these cells in the maintenance as well as the regeneration of the IVD. The Cre/LoxP system offers the convenience of a site‐specific recombinase technique to carry out gene targeting in the DNA of the cells that express the gene driving Cre. The tamoxifen (Tam) mediated induction of Cre in the modified *CreER*, or *CreERT2* transgene offers the additional advantage to regulate recombination of the target gene with time, and in specific tissues. However, despite the innovative and technological advancements in mouse genetic approaches, the application of such robust genetic approaches in IVD biology and disease had been modest. The currently available driver lines to target conditional alleles in the NP cells are the non‐inducible *Noto* ^*Cre*^ (McCann et al., 2012), *Shh* ^*Cre*^ (Harfe et al., 2004), and *Foxa2* ^*Cre*^ (Uetzmann, Burtscher, & Lickert, 2008) alleles, or Tam‐inducible *Agc1* ^*CreERT2*^ (Henry et al., 2009), *Col2a1* ^*CreERT2*^ (Chen et al., 2007), and *Col2a1* ^*CreER*^ (Nakamura, Nguyen, & Mackem, 2006). And, the *Noto* ^*Cre*^ (Bedore et al., 2013; McCann et al., 2012), *Shh* ^*Cre*^ (Winkler, Mahoney, Sinner, Wylie, & Dahia, 2014; Wu et al., 2013), *Foxa2* ^*Cre*^ (Merceron et al., 2014; Uetzmann et al., 2008), *Agc1* ^*CreERT2*^ (Alkhatib et al., 2018; Alvarez‐Garcia et al., 2018; Henry et al., 2009; Liao et al., 2019; Novais, Diekman, Shapiro, & Risbud, 2019), and *Col2a1* ^*CreERT2*^ (Chen et al., 2007; Zheng et al., 2018) alleles have been used to understand the development, regulation, and aging of the mouse NP cells in the IVD. However, none of these driver lines are appropriate to target NP cells during the postnatal stages due to the constraint of spatial and temporal regulation of their action, and specificity to the NP cells. Hence, there is a caveat in their application to understand the role of NP cells in postnatal IVD growth and maintenance. Therefore, development or characterization of an inducible and efficient driver line to target specific components of the IVD is required. Moreover, an ideal driver line would maintain expression for the entirety of the mouse lifespan, thereby allowing for investigation past skeletal maturity and during aging for research aimed at understanding IVD aging, degeneration, as well as regeneration. Considering the role of NP cells in postnatal IVD maintenance is an essential area of research related to IVD biology and of interest for IVD regeneration, it is imperative to establish and characterize an efficient and inducible driver line to target the NP cells.

Cytokeratin 19 (*Krt19, Ck19, or K19*) is a member of the keratin family of proteins. There are two groups of keratins: cytokeratin and hair keratin. The cytokeratins are intermediate filament proteins that are part of the cytoskeleton and crucial for the structural integrity of all epithelial cells. *Krt19 is* expressed by the notochord, the precursor of NP cells. Previously *CK19* ^*Cre*^ was used to target *Sox9* conditional allele to understand the role of *Sox9* in the mouse notochord (Barrionuevo, Taketo, Scherer, & Kispert, 2006). Expression of KRT19 has been reported in the NP cells of various model systems including rat (Lee et al., 2007), bovine (Minogue, Richardson, Zeef, Freemont, & Hoyland, 2010), human (Minogue et al., 2010; Weiler et al., 2010), and mouse (Dahia, Mahoney, & Wylie, 2012). Also, KRT19 is considered a molecular marker of NP cells (Risbud et al., 2015). Results from previous studies showed that all NP cells of neonatal mouse lumbar IVDs are positive for KRT19 immunostaining (Dahia et al., 2012). Moreover, NP cells from a year‐old mouse continue to express *Krt19* messenger RNA (mRNA), although its expression declined with age (Winkler et al., 2014). A Tam‐inducible Cre constructed under the promoter of *Krt19* is available (also known as *CK19* ^*CreERT*^ and *Krt19* ^*tm1(cre/ERT)Ggu*^/J; Means, Xu, Zhao, Ray, & Gu, 2008). Hence, this study aims to characterize the *Krt19* ^*CreERT*^ allele for Tam‐induced recombination in the NP cells using conditional fluorescent reporter alleles in postnatal mice at an early neonatal stage, at skeletal maturity, middle age, and by 2 years of age. The postnatal stages analyzed for characterization of the *Krt19* ^*CreERT*^ allele are based on the current literature on mouse model system to understand the role of NP cells in the postnatal IVD (Alkhatib et al., 2018; Alvarez‐Garcia et al., 2018; Bonavita et al., 2018; Dahia et al., 2012; Vincent et al., 2019; Wu et al., 2013).

## 2 METHODS

### 2.1 Mice

All mice used in the study were maintained in adherence with the guidelines delineated by the National Institutes of Health Guide for the Care and Use of Laboratory Animals. The experiments were conducted following approval and in adherence to institutional guidelines under Institutional Animal Care and Use Committee at Weill Cornell Medical College. Up to four littermates were housed together in a cage in a 12‐hr day and 12‐hr night cycle facility with food and water *ad libitum. Krt19* ^*tm1(cre/ERT)Ggu*^ *(Krt19* ^*CreERT*^ or *CK19* ^*CreERT*^, [Means et al., 2008]), *Gt(ROSA)26Sor* ^*tm3(ACTB‐tdTomato,‐EGFP)Luo*^ */J* (*R26* ^*mT/mG*^, [Muzumdar, Tasic, Miyamichi, Li, & Luo, 2007]), and *Gt(ROSA)26Sor* ^*tm14(CAG‐tdTomato)Hze*^/J (*R26* ^*tdTOM*^, [Madisen et al., 2010]) mice lines were used in the study. The genotypes were confirmed by polymerase chain reaction using genomic DNA from the toe or tail biopsy of neonatal mice, and using allele‐specific primers as previously published (Madisen et al., 2010; Means et al., 2008; Muzumdar et al., 2007). Tamoxifen (T5648; Sigma‐Aldrich) was prepared in warm corn oil (20 mg/ml) and dissolved by overnight incubation in a shaker at 37°C. Previous studies show rapid and robust recombination following oral gavage of tamoxifen in adult mice (Park et al., 2008). Also, repeated intraperitoneal injections could result in sterile peritonitis due to the accumulation of oil in the peritoneal cavity of the mice (Whitfield, Littlewood, & Soucek, 2015). Hence, for skeletally mature mice tamoxifen was administered by oral gavage at a dose of 200 µg/g body weight (Bowers et al., 2012; Lapinski et al., 2007; Lapinski et al., 2012), three times and 1 day apart. In neonates, tamoxifen was injected subcutaneously or intraperitoneally at a dose of 200 µg/g body weight using 31‐gauge syringe needle either every day or every other day. Phosphate buffered saline (PBS) was injected intraperitoneally at P26 to *Krt19* ^*CreERT/+*^; *R26* ^*tdTOM*^ as no tamoxifen control.

### 2.2 Genetic recombination and immunofluorescence analysis

The entire spine and hind‐limbs were dissected in cold PBS and fixed for 4 hours in 4% paraformaldehyde, and then washed in PBS for imaging, or were processed for cryosectioning. The spine and knee joints from 6 M old *Krt19* ^*CreERT/+*^; *R26* ^*tdTOM*^ mice were imaged using DsRed Zeiss Axio Zoom.V16 stereoscope, DsRed epifluorescence, and accompanying Zen software. The samples from skeletally mature mice decalcified for 9 days in 0.5 ethylenediaminetetraacetic acid (E9884; Sigma‐Aldrich), pH 7.6 at 8°C. Following decalcification, all samples were washed three times for 30 min each in cold PBS. Next, the spines were prepared for cryosectioning using Tissue‐Tek^®^ optimum cutting temperature (102094‐106; O.C.T, VWR) molds, and were snap frozen and stored at −80°C until further use. Cryosections were prepared at a thickness of 8 µm using a Leica cryostat and stored at −80°C. The spine was cryosectioned in the coronal plane and transverse plane, while the knee joint, was cryosectioned in the sagittal plane. For determining recombination efficiency, the cryosections were washed twice in PBS and counterstained with 4′,6‐diamidino‐2‐phenylindole (DAPI; 1:5,000; D1306; Life Technologies) for 5 min. The slides were mounted using ProLong^TM^ Diamond (P36962; Life Technologies), and imaged using DAPI, GFP, and TxRD filter cubes of Nikon Eclipse microscope and NIS elements software (Nikon, Japan). Images were captured at 10× and 60× magnifications. High magnification (60×) images were deconvoluted in the NIS elements software using the Landweber algorithm and with 15 iterations.

Immunostaining was conducted on the IVDs cryosectioned in the coronal plane as previously described (Dahia et al., 2012). Briefly, first, the cryosections were air‐dried and rehydrated in PBS. Sections were permeabilized for 20 min using 0.25% Triton‐x100 prepared in PBS. Next, the sections were blocked for 1 hour at room temperature in blocking buffer (10% donkey serum [017‐000‐121; Jackson ImmunoResearch], 4% immunoglobulin G [IgG]‐free bovine serum albumin [001‐000‐162; Jackson ImmunoResearch] and 0.1% PBST) in a humidified chamber. Then the samples were incubated with KRT19 primary antibody (KRT19/TROMA‐III), rat monoclonal IgG (1:100; DSHB, TROMA‐III) prepared in blocking buffer overnight at 4°C and in a humidified chamber. The next day slides were washed in PBS three times and for 5 min each. Then the slides were incubated with Alexa Fluor^®^ 647‐AffiniPure goat anti‐rat IgG (112–605‐062; Jackson ImmunoResearch) secondary antibody diluted in blocking buffer, in a room temperature humidifier for 1 hour and protected from light. Sections were counterstained with DAPI (1:5,000; D1306; Life Technologies) for 5 min and mounted in ProLong™ Gold (P36934; Life Technologies). A negative control slide was also processed along with the samples and in a similar way except for not incubating with primary antibody. This negative control slide was used to determine background and exposure parameters. All samples were imaged at 10× and 60× magnifications as described above. High magnification (60×) images were deconvoluted as described above.

### 2.3 Quantification of immunofluorescence data

**Figure 1:**
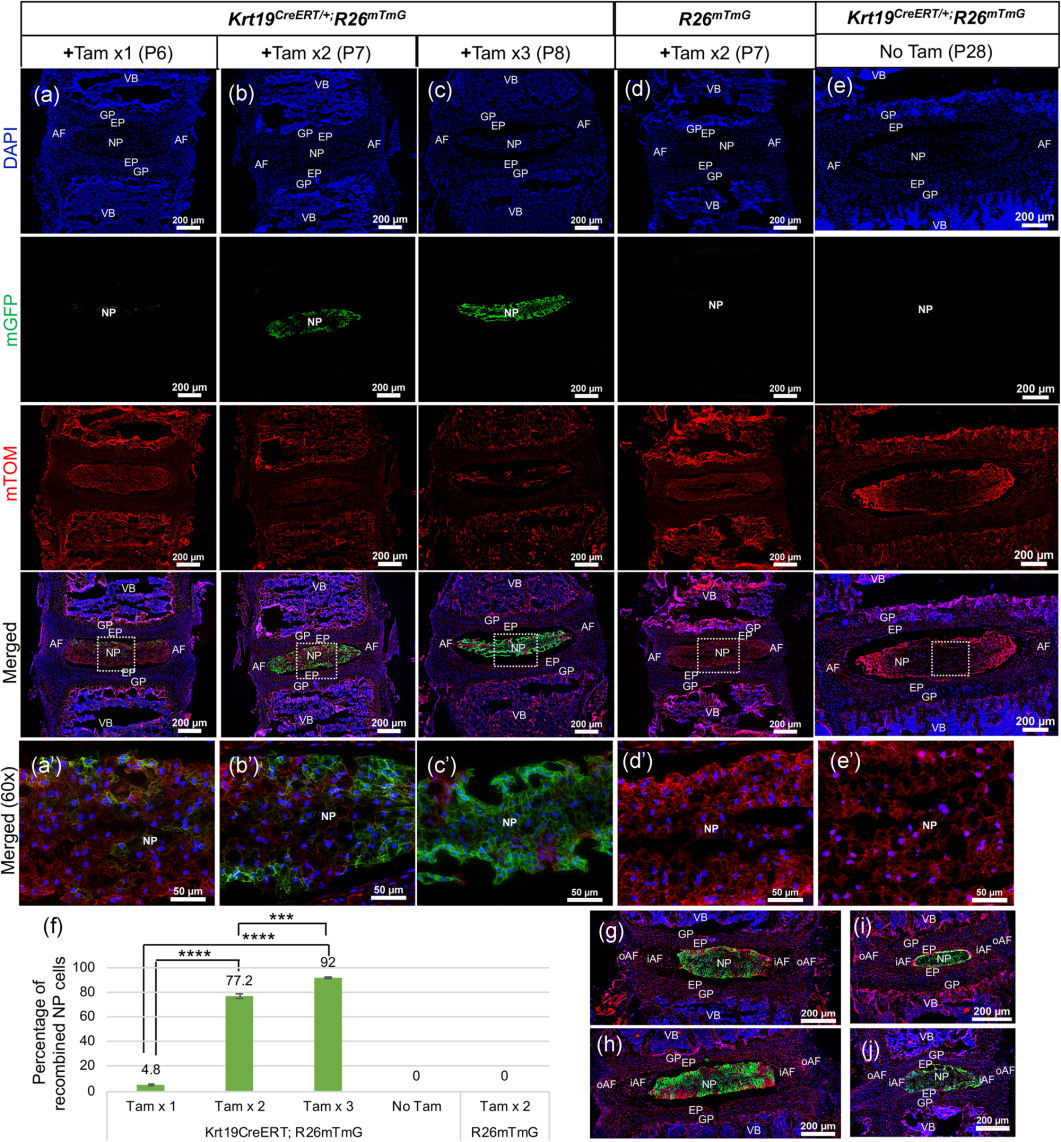
Efficient targeting of the neonatal mouse NP cells by *Krt19^CreERT^* allele is dose and time dependent. Representative epifluorescence images of lumbar IVDs cryosectioned in the mid‐coronal plane from P6 to P28 old mice. (a,a’) Lumbar IVD from P6 *Krt19^CreERT/+^; R26^mT/mG^* following one dose of tamoxifen (+Tam ×1). (b,b’) Lumbar IVD from P7 *Krt19^CreERT/+^; R26^mT/mG^* following two doses of tamoxifen (+Tam x2). (c,c’) Lumbar IVD from P8 *Krt19^CreERT/+^; R26^mT/mG^* following three doses of tamoxifen (+Tam ×3). (d,d’) Lumbar IVD from P7 *R26^mT/mG^* following two doses of tamoxifen (+Tam ×2). (e,e’) Lumbar IVD from P28 *Krt19^CreERT/+^; R26^mT/mG^* mice following PBS treatment (No Tam control). (f) Quantification of the percentage of recombined NP cells calculated over a total number of NP cells in the disc. Epifluorescence images of the mid‐coronal section of the cervical (g), thoracic (h), sacral (i), and caudal (j) IVDs from P7 *Krt19^CreERT/+^; R26^mT/mG^* following two doses of tamoxifen treatment (+Tam ×2). All cryosections were counterstained with DAPI (blue). Images in (a–e,g–j) are captured at 10x magnification. Images in (a’–e’) are captured at 60x magnification. For testing recombination, *n* = 2 for +Tam ×1, *n* = 2 +Tam ×2, *n* = 3 +Tam x3, *n* = 4 +Tam ×2 for R26mT/mG only, and *n* = 2 for PBS only and no Tam control. ***<0.001 ****<0.0001. One‐way ANOVA with post hoc Tukey test. AF, annulus fibrosus; ANOVA, analysis of variance; DAPI, 4',6‐diamidino‐2‐phenylindole; EP, endplate; GP, growth plate; iAF, inner annulus fibrosus; IVD, intervertebral disc; Krt19, keratin 19; NP, nucleus pulposus; oAF, outer annulus fibrosus; Tam, tamoxifen; VB, vertebral body; x, times [Color figure can be viewed at wileyonlinelibrary.com]

**Figure 2:**
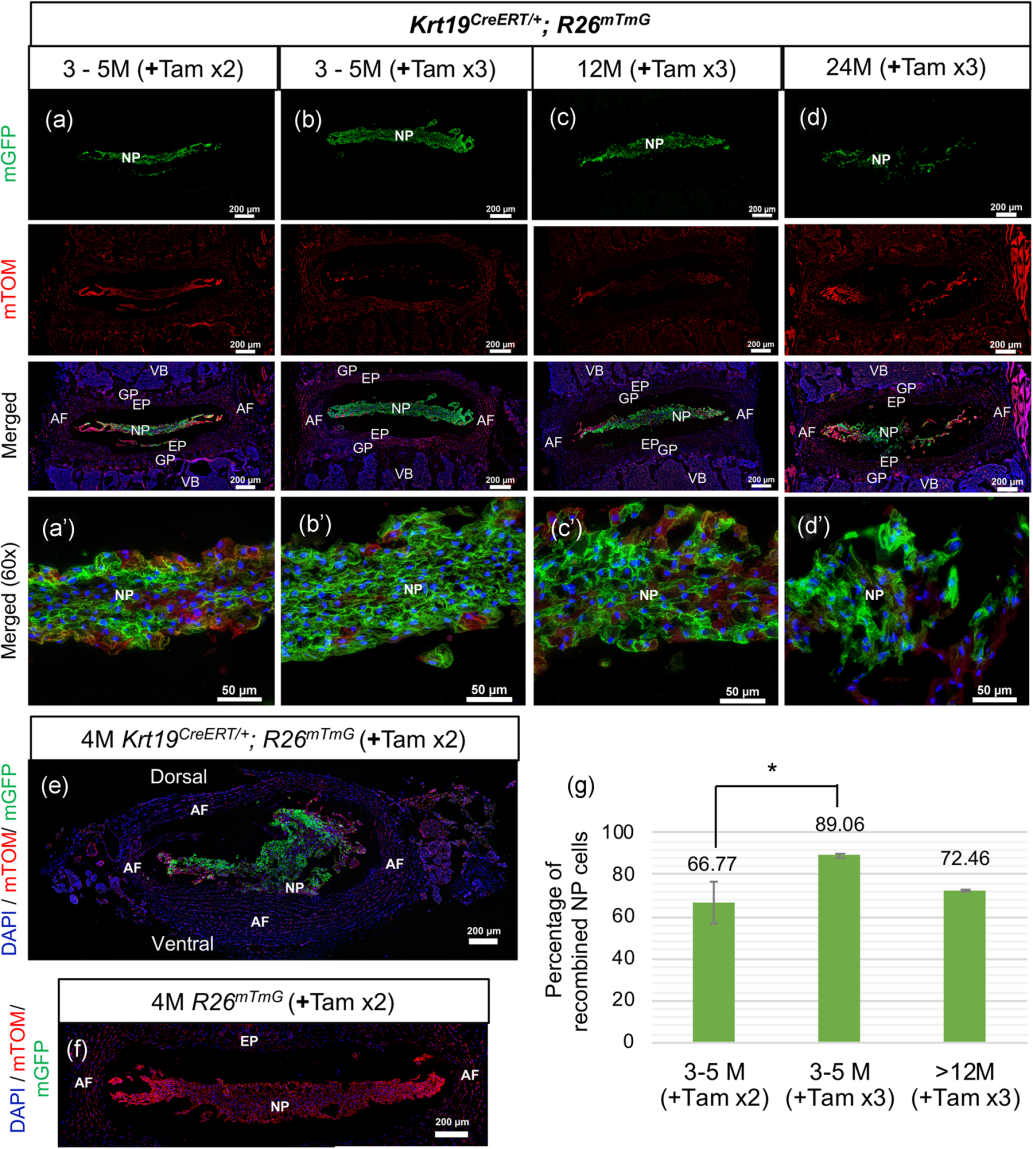
Efficient and specific targeting of the NP cells by *Krt19^CreERT^* allele in the IVD with age. (a–d’,f) Representative epifluorescence images of mid‐coronal lumbar IVDs from adult mice at different ages. Lumbar IVDs from 3–5 M (a–b’), 12 M (c,c’), and 24 M old (d,d’) tamoxifen‐treated *Krt19^CreERT/+^; R26^mT/mG^* mice. (e) Representative epifluorescence image of lumbar IVD cryosectioned in the transverse plane from 4 M old tamoxifen‐treated *Krt19^CreERT/+^; R26^mT/mG^* mouse. (f) Representative epifluorescence image of the mid‐coronal cryosection from 4 M old tamoxifen‐treated *R26^mT/mG^* mouse. (g) Quantification of percentages of mGFP+NP cells calculated over a total number of NP cells. All cryosections were counterstained with DAPI (blue). (a–f) Images captured at 10x magnification. (a’–d’) Images captured at 60× magnification. For testing recombination, *n* = 3 for +Tam ×2 (a,a’,e), *n* = 4 +Tam ×3 (b,b’), *n* = 3 +Tam ×3 over 12 M of age (c–d’), *n* = 3 +Tam ×2 for R26mT/mG only (f). *<0.05. One‐way ANOVA with post hoc Tukey test. AF, annulus fibrosus; ANOVA, analysis of variance; DAPI, 4',6‐diamidino‐2‐phenylindole; EP, endplate; GP, growth plate; IVD, intervertebral disc; Krt19, keratin 19; NP, nucleus pulposus; Tam, tamoxifen; VB, vertebral body; x, times [Color figure can be viewed at wileyonlinelibrary.com]

**Figure 3:**
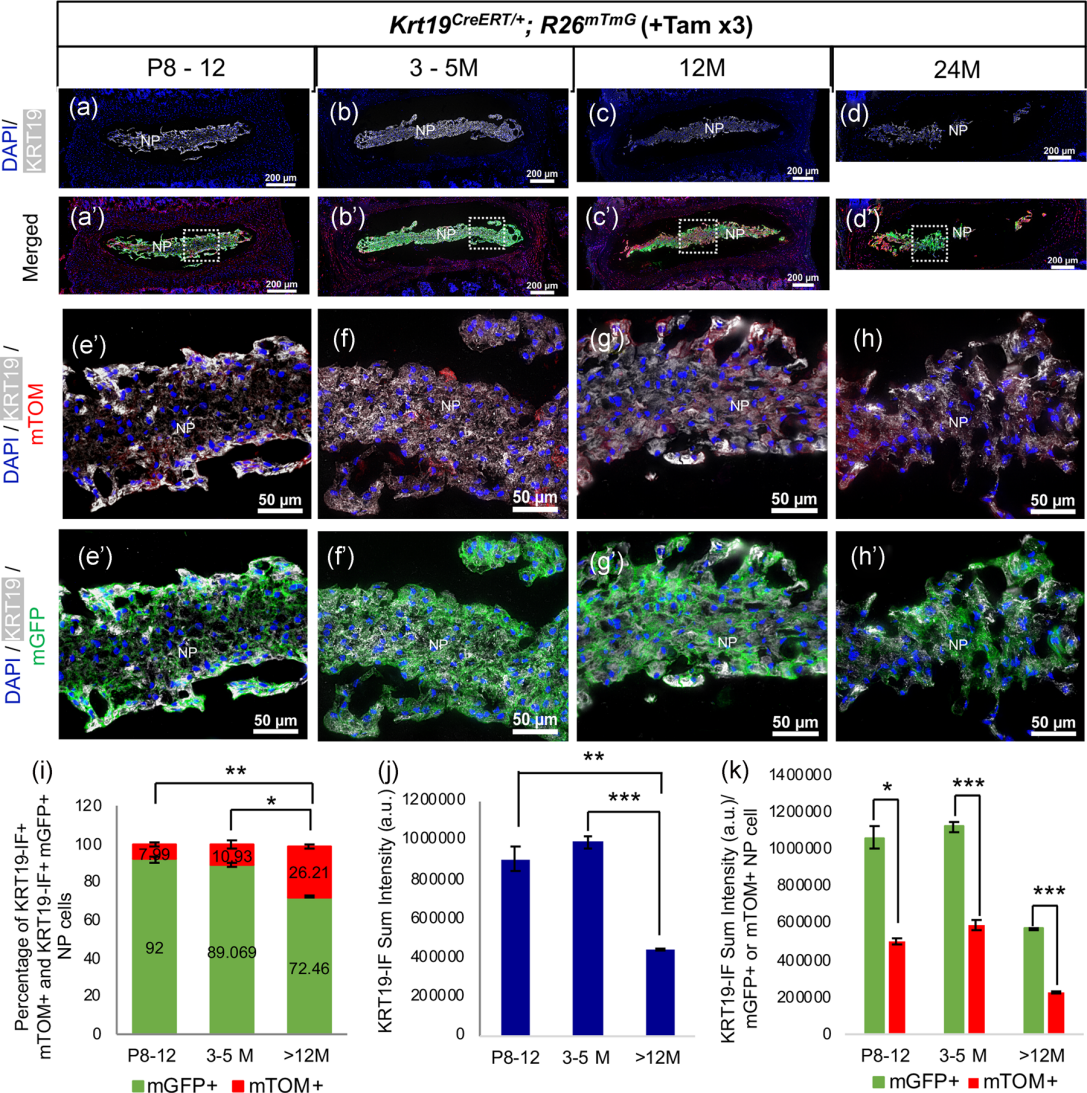
Immunostaining for KRT19 expression in the NP cells with age. (a–h’) Representative epifluorescence images of the mid‐coronal cryosections of lumbar IVDs immunostained for KRT19 (white). Lumbar IVDs from P8–12 (a,a’,e,e’), 3–5 M (b,b’,f,f’), 12 M (c,c’,g,g’), and 24 M old (d,d’,h,h’) tamoxifen‐treated *Krt19^CreERT/ +^ ; R26^mT/mG^* mice. (e–h) Colocalization of KRT19‐ IF+ (white) and mTOM+ (red) in the NP cells imaged at 60x magnification. (e’–h’) Colocalization of KRT19‐IF+ (white) and mGFP+ (green) in NP cells imaged at 60× magnification. (i) Quantification of the percentages of KRT19‐IF+/mGFP+NP cells and KRT19‐IF+/mTOM+NP cells with age. (j) Quantification of the sum intensity for KRT19 immunofluorescence in the entire NP cell population with age. (k) Quantification of the sum intensity for KRT19 immunofluorescence specifically in the mGFP+NP cells and mTOM+NP cells with age. (a–d’) Images captured at 10× magnification. *n* = 3 for P8–12, *n* = 3 for 3–5 M, *n* = 3 for over 12 M of age. *<0.05, **<0.01. One‐way ANOVA with post hoc Tukey test. ANOVA, analysis of variance; a.u., arbitrary units; IF, immunofluorescence; IVD, intervertebral disc; Krt19, keratin 19; NP, nucleus pulposus; Tam, tamoxifen; x, times [Color figure can be viewed at wileyonlinelibrary.com]

Recombination efficiency was quantified using the NIS elements software. The nuclei were counted by defining a polygonal region of interest (ROI) around the entire NP area and then thresholding for DAPI for object count. Next, the number of mGFP+ or the number of mTOM+ NP cells within that ROI were counted manually, and these numbers were divided by the total number of nuclei to determine the percentage of recombined NP cells. As all NP cells were KRT19 immunofluorescence (IF+), the percentage of mGFP+ and mTOM+ NP cells that expressed KRT19 was calculated as described above. To determine the age‐related change in KRT19 expression in the NP cells, the ROI was drawn around the entire NP area and the sum intensity for KRT19‐IF was determined, which was then divided by the number of cells. To determine the change in KRT19‐IF intensity per NP cell in the recombined (mGFP+) and non‐recombined (mTOM+) NP cell population, a ROI was drawn around 20 individual cells that were either mGFP+ or mTOM+ within a disc. Next the sum intensity for KRT19‐IF within these NP cells was determined and divided by 20 to then determine the average KRT19 intensity per NP cell in each category. Quantification was performed on at least three serial sections, which were considered as technical replicates from each lumbar IVD of a given biological replicate, and the data is presented as the mean ± standard error of the mean (*SEM*). The percentages of recombined NP cells were statistically analyzed using one‐way analysis of variance using GraphPad prism version 8.0. The relevant factor in Figure 1 was the number of doses of Tam that were administered, while age was the relevant factor in Figures 2 and 3. A significant main effect of either factor prompted Tukey post hoc analyses for multiple comparisons. A *p* < .05 was considered statistically significant. To quantify data presented in Figure 3k a two‐tailed *t* test was performed to compare the sum intensity for KRT19‐IF in mGFP+ versus mTOM+ NP cells within each age cohort.

## 3 RESULTS

To determine the specificity and efficiency of *Krt19* ^*CreERT*^ allele for targeting the NP cells of postnatal mouse IVDs, first, we utilized the *R26* ^*mT/mG*^ reporter line. The *R26* ^*mT/mG*^ is a membrane‐tagged dual fluorescent (tomato and GFP) Cre*‐*reporter allele (Muzumdar et al., 2007). In the absence of Cre recombinase, all cells and tissues of the mouse carrying the *R26* ^*mT/mG*^ allele express the membrane‐bound tdTomato (mT or mTOM) and appear red under epifluorescence. However, in the presence of Cre under a cell‐specific promoter, recombination will cause floxing out, or targeting, the mTOM cassette, and induce the expression of membrane‐bound EGFP (mG or mGFP) and now these cells will appear green under epifluorescence (Muzumdar et al., 2007). To analyze the specificity of *Krt19* ^*CreERT*^ to target NP cells, we crossed *Krt19* ^*CreERT*^ allele with *R26* ^*mT/mG*^ reporter allele to generate *Krt19* ^*CreERT/+*^; *R26* ^*mT/mG*^ line. Hence, the *Krt19* ^*CreERT/+*^; *R26* ^*mT/mG*^ line will not only differentiate recombined cells (green, mGFP+) from non‐recombined cells (red, mTOM+) but will also allow identifying each component (NP vs. AF or EP) of the IVD, and enable to precisely measure recombination efficiency (mGFP+/DAPI+).

### 3.1 *Krt19* ^*CreERT*^ allele efficiently targets NP cells in the IVDs of neonatal mice

First, the adequate dose of Tam and the time required to efficiently induce *Krt19* ^*CreERT*^ mediated recombination of the *R26* ^*mT/mG*^ reporter in the NP cells was determined in postnatal day five (P5) pups. Following a single dose of tamoxifen (+Tam ×1) and 24 hr later, only 4.8% (*SEM* ± 0.5) of NP cells were observed to turn mGFP+ in P6 mouse lumbar IVD (Figure 1a,f). Following two doses of Tam administrated at P5 and P6 (+Tam ×2), 77.2% (*SEM* ± 1.813) of NP were mGFP+ in P7 mouse lumbar IVDs (Figure 1b,f). Following three doses of Tam administrated at P5, P6, and P7 (+Tam ×3), 92.0% (*SEM* ± 0.362) of NP cells were mGFP+ in P8 mouse lumbar IVDs (Figure 1c,f). Tam administration at P5 and P6 (+Tam ×2) to the *R26* ^*mT/mG*^ littermates did not cause any nonspecific recombination, and all cells of the lumbar IVD, including NP cells, continued to express mTOM as shown at P7 (Figure 1d,f). Also, PBS treated P28 *Krt19* ^*CreERT*^; *R26* ^*mT/mG*^ mice that served as no tam control (no Tam), did not show any leaky recombination in the NP cells, and all cells in the IVD continued to be mTOM+ (Figure 1e,f). At all doses tested and time points analyzed, the mGFP+ cells were restricted only to the NP region of the lumbar IVD (Figure 1a–c). Next, the Tam‐induced *Krt19* ^*CreERT*^ mediated recombination of *R26* ^*mT/mG*^ reporter was analyzed in the IVDs from the cervical, thoracic, sacral, and caudal regions of the spine following Tam administration at P5 and P6 (+Tam ×2). NP cells exclusively turned mGFP+ in the cervical (Figure 1g), thoracic (Figure 1h), sacral (Figure 1i), and caudal (Figure 1j) IVDs at P7, indicating that *Krt19* ^*CreERT*^ specifically targets NP cells irrespective of the region of the spine in a week‐old mouse. No mGFP+ cells were observed in the other components of IVD throughout the spine including the inner region of AF (iAF), the outer region of AF (oAF), and the cartilaginous EP with all doses and time points tested (Figure 1a–j). Also, the adjacent growth plate (GP), and vertebral bodies (bone as well as marrow) of the Tam‐treated *Krt19* ^*CreERT/+*^; *R26* ^*mT/mG*^ pups were negative for any recombination (Figure 1a–j). The results indicate that the *Krt19* ^*CreERT*^ mediated recombination is specific to NP cells within the spine of a neonatal mouse.

### 3.2 *Krt19* ^*CreERT*^ allele efficiently targets NP cells in the IVDs of skeletally mature mice

Next, the Tam‐induced *Krt19* ^*CreERT*^ mediated recombination was evaluated in the NP cells of skeletally mature, and aged mice. First, the dosage of Tam required to yield high recombination was determined. Tam was administered by oral gavage to three‐month‐old to five‐month‐old (3–5 M) *Krt19* ^*CreERT/+*^; *R26* ^*mT/mG*^ mice. Considering IVD is avascular with a slower rate of diffusion with age (Urban & Winlove, 2007), another dose of tamoxifen was administered a day later (+Tam ×2). Two days after the last dose of Tam, lumbar spine was dissected and cryosectioned. Images were captured using a wide‐field epifluorescence microscope at low and high magnifications (Figure 2a,). Quantification of mGFP+ NP cells revealed that *Krt19* ^*CreERT*^ mediated recombination of the *R26* ^*mT/mG*^ allele occurred in 66.77% (*SEM* ± 9.821) of the NP cells (Figure 2a,g). Administration of a third dose of tamoxifen (+Tam ×3), increased the recombination to 89.06% (*SEM* ± 0.870) in the NP cells of 3–5 M old *Krt19* ^*CreERT/+*^; *R26* ^*mT/mG*^ mice (Figure 2b,g). Finally, Tam‐induced *Krt19* ^*CreERT*^ mediated recombination of *R26* ^*mT/mG*^ allele was assessed in 12 M and 24 M old mice. Three oral gavages of tamoxifen (+Tam ×3) were administered that were 1 day apart. Tam‐induced recombination was observed in 72.46% (*SEM* ± 0.662) of the NP cells in the IVDs from 12 M and 24 M old *Krt19* ^*CreERT/+*^; *R26* ^*mT/mG*^ mice (Figure 2c,d,g). All other tissues in the spine including inner and outer AF, cartilaginous EP, growth plate, and vertebral bodies (bone as well as marrow) continued to be mTOM+ at all ages analyzed in the Tam‐induced *Krt19* ^*CreERT/+*^; *R26* ^*mT/mG*^ mice (Figure 2a–d). A transverse section through the lumbar IVD of Tam‐induced (+Tam ×2) 4 M old *Krt19* ^*CreERT/+*^; *R26* ^*mT/mG*^ mice also shows that *Krt19* ^*CreERT*^ is specific to NP cells that were mGFP+, while the surrounding AF did not undergo recombination and continued to be mTOM+ (Figure 2e). Figure 2f shows the absence of recombination in a 4 M old Tam‐treated (+Tam ×2) *R26* ^*mT/mG*^ littermate and all cells in the lumbar spine, including NP cells, were mTOM+. Also, no recombination or mGFP+cells were observed in Tam‐treated *R26* ^*mT/mG*^ control mice analyzed at 12 M and 24 M of age (data not shown). These results indicate that the *Krt19* ^*CreERT*^ allele is specific to NP cells in the spine at all ages analyzed (P5 through 24 M). Also, depending on the age of the mouse, three doses of tamoxifen to *Krt19* ^*CreERT*^ mice can achieve about 72.46–92% recombination.

### 3.3 Marginally reduced efficiency in adult mice is not due to complete loss of KRT19 expression

Given that only 72.46% of the NP cells in aged mice (12 M+) showed recombination, compared to over 92% recombination observed in the NP cells from younger mice, we reasoned that the lower efficiency could be due to loss of KRT19 expression in a subset of NP cells with age. The lumbar IVDs from Tam‐treated *Krt19* ^*CreERT/+*^; *R26* ^*mT/mG*^ mice that were P8–12, 3–5 M, 12 M, and 24 M of age were immunostained for KRT19. Low and high magnification images were captured to determine the localization of KRT19 in NP cells that were either mGFP+ or mTOM+ (Figure 3a–h). Immunofluorescence for KRT19 (KRT19‐IF) shows that it is ubiquitously expressed by all NP cells and co‐localizes indiscriminately with both the mGFP+ and mTOM+ NP cells at all ages analyzed (Figure 3a–d,i). Although at high magnification, the intensity for KRT19‐IF is not uniform amongst the NP cells (Figure 3e–h), however, all NP cells continued to be KRT19‐IF+ (Figure 3i). Quantification of the KRT19‐IF+ NP cells that were either mGFP+ or mTOM+ shows that the proportion of KRT19‐IF+ NP cells that are mGFP+ reduces with age (Figure 3i). A significant decrease (*p* < .01) in KRT19‐IF sum intensity was observed in the NP cells from P8 to over one‐year of age (Figure 3j). Next, a significant (*p* < .05) decline in KRT19‐IF sum intensity in the non‐recombined (mTOM+) NP cells versus recombined (mGFP+) was observed in the NP cells of P8–12, which further declined after 3 months of age (Figure 3k). Results suggest that the reduced recombination in aged mice is likely due to reduced expression of KRT19 by non‐recombined cells, causing insufficient induction of Cre in these subsets of NP cells. Also, possibly due to reduced diffusion of tamoxifen into the NP cells of the aged IVDs, or a combination of the above.

### 3.4 Validation of efficiency and specificity of Krt19^*CreERT*^ to target the NP cells using an additional independent reporter allele

**Figure 4:**
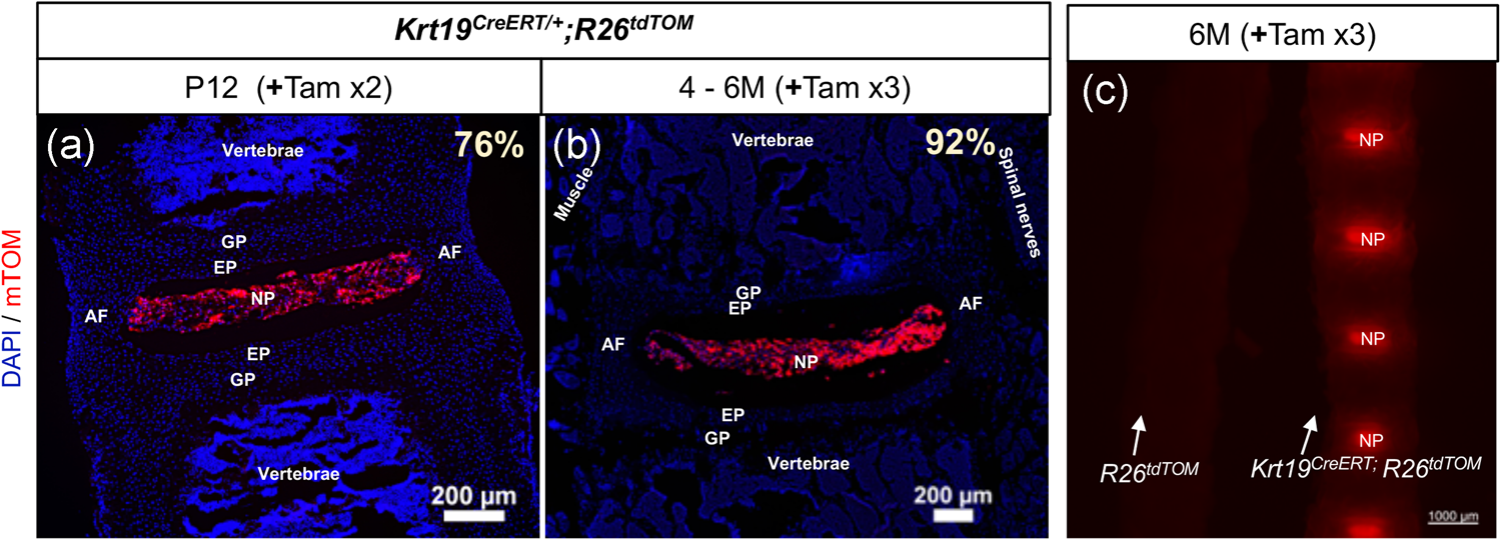
Efficient and specific targeting of the *R26^tdTOM^* allele by *Krt19^CreERT^* in the NP cells. (a,b) Representative epifluorescence images of the mid‐coronal cryosections from the lumbar IVDs of P12 and 4–6 M old tamoxifen‐treated *Krt19^CreERT/+^; R26^tdTOM^* mice. Sections were counterstained with DAPI (blue) and imaged at 10× magnification. Numbers in (a) and (b) represents the percentage of recombined (tdTOM+) NP cells. (c) Representative images of the thoracic spines from 6 M old tamoxifen‐treated *R26^tdTOM^* only (left) and *Krt19^CreERT/+^; R26^tdTOM^* (right) mice imaged together using DsRed epifluorescence and stereoscope. For testing recombination, *n* = 2 at P12 (a), *n* = 3 at 4–6 M (b) of age. AF, annulus fibrosus; DAPI, 4',6‐diamidino‐2‐phenylindole; EP, endplate; GP, growth plate; iAF, inner annulus fibrosus; IVD, intervertebral disc; Krt19, keratin 19; NP, nucleus pulposus; oAF, outer annulus fibrosus; Tam, tamoxifen; VB, vertebral body; x, times [Color figure can be viewed at wileyonlinelibrary.com]

Next, to validate the specificity of *Krt19* ^*CreERT*^ for the NP cells in the spine, we tested the Tam‐induced *Krt19* ^*CreERT*^ mediated recombination using a different conditional reporter allele. Towards this, *Krt19* ^*CreERT*^ was crossed with Ai14 (*R26* ^*tdTOM*^) to generate the *Krt19* ^*CreERT/+*^; *R26* ^*tdTOM*^ line. The Ai14 is a Cre‐reporter allele having a *loxP‐*flanked STOP cassette that prevents the expression of red fluorescent protein variant (tdTomato or tdTOM; Madisen et al., 2010). Following Cre or Tam‐induced CreERT mediated recombination, robust cytoplasmic tdTOM fluorescence can be analyzed in time and cell‐specific manner. Following Tam administration at P7 and P9 (+Tam ×2) to *Krt19* ^*CreERT/+*^; *R26* ^*tdTOM*^ mice, 76% of the NP cells were tdTOM+ in the P12 lumbar IVD (Figure 4a). Next, following three doses of tamoxifen (+Tam ×3) administration to 4–6 M old *Krt19* ^*CreERT/+*^; *R26* ^*tdTOM*^ mice, 92% of the NP cells were tdTOM+ (Figure 4b). The tdTOM+ cells were observed only in the NP cells of the IVD at both the ages analyzed. The cells in the surrounding tissues of the spine including muscle, spinal nerves, vertebral bodies, GP, EP, and AF did not undergo recombination and were negative for tdTOM epifluorescence (Figure 4a,b). In another experiment, 6 M old *R26* ^*tdTOM*^ and *Krt19* ^*CreERT/+*^; *R26* ^*tdTOM*^ littermates were Tam‐treated (+Tam ×3). The thoracic spine from both the mice was dissected and imaged using DsRed epifluorescence and stereomicroscope (Figure 4c). Only the nucleus pulposus from Tam‐treated *Krt19* ^*CreERT/+*^; *R26* ^*tdTOM*^ mouse were tdTOM+, and no epifluorescence was detected form the Tam‐treated *R26* ^*tdTOM*^ littermate. These results independently confirm that *Krt19* ^*CreERT*^ is an efficient driver for conditional targeting of postnatal mouse NP cells.

### 3.5 *The Krt19* ^*CreERT*^ allel is specific to the NP cells in the musculoskeletal system

**Figure 5:**
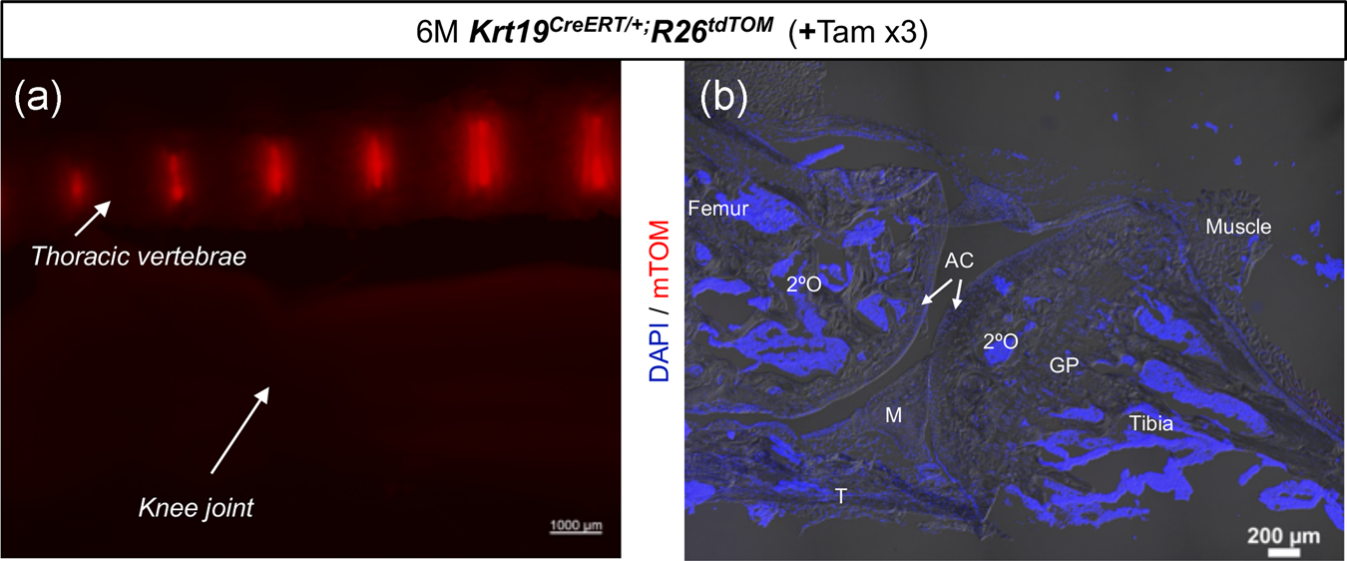
The *Krt19^CreERT^* driver line is specific to the NP cells and does not target other musculoskeletal tissues analyzed. (a) Representative stereoscopic image using DsRed epifluorescence of the thoracic spine (top) and knee joint (bottom) from 6 M old tamoxifen‐treated *Krt19^CreERT/+^; R26^tdTOM^* mouse. (b) Representative epifluorescence image of sagittal cryosection of the knee joint from 6 M old Tam‐treated *Krt19^CreERT/+^; R26^tdTOM^* counterstained with DAPI (blue) and captured at 10× magnification. *n* = 2. AC, articular cartilage; DAPI, 4',6‐diamidino‐2‐phenylindole; GP, growth plate; Krt19, keratin 19; M, meniscus; NP, nucleus pulposus; 2°O, secondary center of ossification; Tam, tamoxifen; T, tendon; x, times [Color figure can be viewed at wileyonlinelibrary.com]

When designing experiments to determine the effect of genetic manipulation in the NP cells of the IVD, it is crucial that cells from other musculoskeletal tissues including joints are not targeted, especially considering that the mouse is a quadruped and defects in the joints may, in turn, affect the spine and the IVDs. Hence, following administration of three doses of Tamoxifen (+Tam ×3), the *Krt19* ^*CreERT/+*^ mediated recombination of *R26* ^*tdTOM*^ allele was analyzed in the knee joint of 6 M old mice. Figure 5a shows the thoracic spine and knee joint dissected from the same mouse and imaged together using DsRed epifluorescence and stereomicroscope. Only the NP in the spine was observed to be tdTOM+. No fluorescence signal was detected from the knee joint. Figure 5b shows the DAPI stained sagittal section of the knee joint imaged using a wide‐field epifluorescence microscope equipped with darkfield imaging. No tdTOM+ cells were observed in the entire knee joint. All cells and tissues of the knee joint including tibia, femur, articular cartilage (AC), the secondary center of ossification (2°O), growth plates (GP) of these long bones, meniscus (M), tendon (T), and muscle were negative for tdTOM epifluorescence. These results indicate that the *Krt19* ^*CreERT*^ is exclusive to the NP cells amongst the various musculoskeletal tissues analyzed in the current study.

## 4 DISCUSSION

Results of the current study demonstrate that Tam induction‐mediates *Krt19* ^*CreERT*^ to specifically target the conditional fluorescent alleles in the NP cells of the postnatal mouse spine. Usually, high recombination is observed 48 hr following Tam treatment in vascularized tissues. However, as IVDs are large and avascular, molecules from circulatory system slowly diffuses through the growth plate and EP towards the NP cells (Urban & Winlove, 2007; Wong et al., 2019). Results from the current study show that at least three doses of Tam at a concentration of 200 µg/g body weight are required to achieve about 92% recombination of conditional reporter alleles in the NP cells of neonatal mice. In the current study, Tam‐mediated recombination was tested in the neonatal mice by adminstering Tam on consecutive days (Figure 1b), and with a gap of 1 day (Figure 4a). Both the approaches yielded similar efficiency of recombination for the specific dose of Tam tested. As a relatively higher dose of Tam is required to target the NP cells, if the experimental design does not require immediate analysis, our recommendation is to give at least one‐day gap in between Tam treatments to neonatal mice. Besides, in the absence of Tam, no recombination was observed, suggesting that *Krt19* ^*CreERT*^ allele does not mediate leaky induction in the NP cells. In young adult mice of over 3 months of age, three doses of Tam at a concentration of 200 µg/g body weight was required to induce about 90% recombination in the NP cells of lumbar IVDs (Figures 2b, 4b). However, the same dose of Tam yielded only 72% recombination in the NP cells of mice that were older than 1 year of age. One possible explanation for the relatively lower recombination of *Krt19* ^*CreERT/+*^; *R26* ^*mT/mG*^ mice at 12 M and 24 M of age is that the proportion of *Krt19* expressing NP cells reduces with age. However, immunostaining for KRT19 on the lumbar spine of *Krt19* ^*CreERT/+*^; *R26* ^*mTmG*^ mice at P8–12, 3–5 M, 12 M, and 24 M of age revealed that all NP cells ubiquitously express KRT19, although its expression reduces with age. Also, quantification of KRT19‐IF intensity within recombined (mGFP+) and non‐recombined (mTOM+) NP cells revealed that the non‐recombined cells had lower expression of KRT19 at each age. Previously it was demonstrated that the mRNA expression of *Krt19* declines from P4 to 1 year of age in the NP cells of mouse lumbar IVDs (Winkler et al., 2014), hence this was not analyzed in the current study. Results from previous studies also showed that the cartilaginous EP undergoes mineralization with age, which further reduces the diffusion of systemic molecules into the NP space (Urban & Winlove, 2007). Hence, to enhance the *Krt19* ^*CreERT*^ mediated recombination in the NP cells past 1 year of age, either more than three doses of Tam at a concentration of 200 µg/g body weight or higher doses of tamoxifen may be required. Previously, Tam‐mediated *Krt19* ^*CreERT/+*^; *R26* ^*tdTOM*^ recombination was observed only in a subset of NP cells in the sacral IVD of the 12‐week‐old mouse (Bonavita et al., 2018). While KRT19 was expressed by all NP cells in the sacral IVD of P4 mouse, the percentages of KRT19‐IF+ cells in the sacral IVD reduced from P4 to 12‐weeks of age (Bonavita et al., 2018). Hence, the higher recombination observed in the NP cells of P7 mouse sacral IVD (Figure 1i) in the current study is due to ubiquitous KRT19 expression at this age. Also, in the current study, Tam‐induced *Krt19* ^*CreERT*^ mediated recombination occurred with equal efficiency in the NP cells from the IVDs of the cervical, thoracic, lumbar, sacral, and caudal region of the spine in a neonatal mouse, as KRT19 is ubiquitously expressed by NP cells at this age.

The ability to precisely target NP cells at a given time during the postnatal stages has been a limitation. The current strategy to determine loss of gene function in NP cells is either by using non‐inducible alleles including *Noto* ^*Cre*^ (McCann et al., 2012), *Shh* ^*Cre*^ (Harfe et al., 2004), *or Foxa2* ^*Cre*^ (Uetzmann et al., 2008), where the recombination or targeting of conditional allele occurs at the node and early notochord stage in the embryo, which is before the formation of the IVDs. Moreover, in addition to targeting the notochord, both *Shh* ^*Cre*^ and *Foxa2* ^*Cre*^ also mediate recombination of conditional alleles in the adjacent floor plate, and several organs during organogenesis. For example, *Foxa2* ^*Cre*^ mediates unregulated recombination in the progenitor of endoderm during embryogenesis, thus targeting most of the cells and organs derived from the endoderm (Uetzmann et al., 2008). And *Shh* ^*Cre*^ mediates unregulated recombination in organs arising from definitive endoderm including the bladder urothelium (Bell et al., 2011), the epithelium of submandibular glands (Szymaniak et al., 2017), the gastrointestinal tract (Mao, Kim, Rajurkar, Shivdasani, & McMahon, 2010), the trachea and esophagus (Kishimoto et al., 2018), the lung epithelium (Kadzik et al., 2014), and the epidermal placode (Levy, Lindon, Harfe, & Morgan, 2005). Therefore, due to the lack of spatial and temporal regulation of *Shh* ^*Cre*^ and *Foxa2* ^*Cre*^ driver lines, they are unsuitable for targeting conditional alleles to determine their function in postnatal NP cells. For example, in a study by Winkler et al. (2014) the effects of targeting WNT signaling on the NP cells of the IVD was analyzed using *Shh* ^*Cre*^; *Wls* ^*flx/flx*^. However, the mutant embryos had developmental defects in lung and were not viable post birth. Thus, the study was limited to only analyzing the effects of loss of *Wls* (*Wntless* or *Evi*) in the notochord descendant NP cells at E18.5 (Winkler et al., 2014). While *Noto* ^*Cre*^ is specific to the node and early notochord, it nevertheless targets conditional genes during development; hence it will not be feasible to differentiate the role of a specific gene during IVD development from that during postnatal IVD growth and maintenance. A Tam‐inducible *Foxa2* ^*CreERT2*^ (also known as *Foxa2* ^*mcm*^) allele is available (Park et al., 2008), but due to its transient expression in the notochord till E9.5 (Choi et al., 2012), it is not suitable for postnatal studies. An inducible *Shh* ^*CreERT2*^ (Harfe et al., 2004) allele is also available. However, very few NP cells were targeted following Tam administration to *Shh* ^*CreERT2*^ allele in the postnatal mouse IVDs (Zheng et al., 2019). In addition to node or notochord specific alleles, the use of Tam‐inducible alleles under the extracellular matrix genes including aggrecan using *Agc1* ^*CreERT2*^ (Henry et al., 2009; Henry, Liang, Akdemir, & de Crombrugghe, 2012), and collagen type 2a1 using *Col2a1* ^*CreERT2*^ (Chen et al., 2007) are used to target NP cells in the postnatal stages (Alkhatib et al., 2018; Alvarez‐Garcia et al., 2018; Chen et al., 2007; Henry et al., 2012; Liao et al., 2019; Novais et al., 2019; Zheng et al., 2018). However, these Tam‐inducible alleles are not specific to a particular compartment of the IVD (NP, inner and outer AF or EP). In addition, these driver lines also mediate recombination of conditional alleles in cartilage including adjacent vertebral growth plate chondrocytes, AC, growth plate of long bone (Cantley et al., 2013; Henry et al., 2012; Hirai, Chagin, Kobayashi, Mackem, & Kronenberg, 2011; Maeda et al., 2007; Nagao, Cheong, & Olsen, 2016; Ono, Ono, Nagasawa, & Kronenberg, 2014; Zhou et al., 2014), tendon and ligaments (Nagao et al., 2016), and other cartilaginous tissues in the body including nasal capsule cartilage (Kaucka et al., 2018). Hence, in experiments utilizing these driver lines alterations or defects in the musculoskeletal tissues and joints may, in turn, affect the posture of the mouse as well as uneven loading of the spine and the IVDs. Therefore, these inducible driver lines driven by genes that are expressed by various cartilaginous tissues are not ideal for targeting the NP cells. Characterization of a *Col1a2* ^*CreERT*^ allele (Bou‐Gharios et al., 1996) in the three‐week‐old mouse IVD showed that this allele is specific for outer AF (Bedore, Quesnel, Quinonez, Seguin, & Leask, 2016). However, in addition to outer AF, *Col1a2* ^*CreERT*^ is also expressed in several cells, including osteoblasts (Bou‐Gharios et al., 1996). Thus, the major caveat of the currently available Tam‐inducible alleles is their lack of specificity, specifically for genetic experiments aimed at understanding the role of NP cells in postnatal IVD development, maintenance, and aging. The *Krt19* ^*CreER*^ allele characterized in the current study, overcomes these limitations because of its spatiotemporal regulation, specifically in the NP cells of the musculoskeletal system.

Nevertheless, despite the utility of the *Krt19* ^*CreERT*^ in efficiently targeting NP cells, Tam‐mediated recombination of *Krt19* ^*CreERT*^ also occurs in the epithelial cells of the postnatal mouse including pancreatic tissue, hepatic epithelial tissues, intestinal epithelial cells, stomach (Means et al., 2008), and hair follicle stem cells (Zito et al., 2014). Hence, in addition to the NP within the IVD, Tam will also induce *Krt19* ^*CreERT*^ mediated recombination in these tissues. Hence, in the experiments using *Krt19* ^*CreERT*^ allele for conditional knockdown or overexpression of the genes of interest, the effects on these organs should be considered. One of the ways is to weigh the mice before Tam administration and at the end of the experiment to determine changes in body weight compared to littermate controls. However, considering that the IVDs are the largest avascular tissues, and spatially distant from these organs, genetic manipulation in these organs having a direct effect on the NP cells is highly unlikely.

In summary, the current study demonstrates that the *Krt19* ^*CreERT*^ allele precisely and efficiently targets NP cells in the IVD of neonatal, skeletally mature and aged mice, without inducing nonspecific recombination in the surrounding tissues within the spine, as well as other musculoskeletal tissues including the knee joints. Thus, findings from the current study yield a promising avenue for the use of *Krt19* ^*CreERT*^ in developing genetic mouse models by conditional gene targeting, and lineage‐tracing studies to exclusively determine the role of NP cells in the postnatal stages and during degeneration and aging. Such studies will advance our understanding about the role of NP cells at the cellular and molecular level in maintenance of the microenvironment of the IVD during the postnatal age; whether loss of NP‐specific signals affects the IVD microenvironment; and whether quiescent NP cells can be targeted for reactivation of the entire disc (Bonavita et al., 2018) aimed toward disc regeneration.

## CONFLICT OF INTERESTS

The authors declare that there is no conflict of interests.

## AUTHOR CONTRIBUTIONS

Conceptualization, resources, supervision, project administration, funding acquisition, data curation, writing‐review and editing, and methodology by C. L. D. Validation, writing an original draft, and visualization by S. M. and C. L. D. Formal analysis by S. M. Investigation by S. M. and R. P. All authors reviewed the manuscript and gave their final approval for submission.

## References

[jcp28952-bib-0001] Alkhatib, B. , Liu, C. , & Serra, R. (2018). Tgfbr2 is required in Acan‐expressing cells for maintenance of the intervertebral and sternocostal joints. JOR Spine, 1, 1. 10.1002/jsp2.1025 PMC6333471 30662980

[jcp28952-bib-0002] Alvarez‐Garcia, O. , Matsuzaki, T. , Olmer, M. , Miyata, K. , Mokuda, S. , Sakai, D. , … Lotz, M. K. (2018). FOXO are required for intervertebral disk homeostasis during aging and their deficiency promotes disk degeneration. Aging Cell, 17, e12800. 29963746 10.1111/acel.12800 PMC6156454

[jcp28952-bib-0003] Antoniou, J. , Steffen, T. , Nelson, F. , Winterbottom, N. , Hollander, A. P. , Poole, R. A. , … Alini, M. (1996). The human lumbar intervertebral disc: Evidence for changes in the biosynthesis and denaturation of the extracellular matrix with growth, maturation, ageing, and degeneration. Journal of Clinical Investigation, 98, 996–1003. 8770872 10.1172/JCI118884 PMC507515

[jcp28952-bib-0004] Barrionuevo, F. , Taketo, M. M. , Scherer, G. , & Kispert, A. (2006). Sox9 is required for notochord maintenance in mice. Developmental Biology, 295, 128–140. 16678811 10.1016/j.ydbio.2006.03.014

[jcp28952-bib-0005] Bedore, J. , Quesnel, K. , Quinonez, D. , Seguin, C. A. , & Leask, A. (2016). Targeting the annulus fibrosus of the intervertebral disc: Col1a2‐Cre(ER)T mice show specific activity of Cre recombinase in the outer annulus fibrosus. Journal of Cell Communication and Signaling, 10, 137–142. 27173473 10.1007/s12079-016-0329-7 PMC4882308

[jcp28952-bib-0006] Bedore, J. , Sha, W. , McCann, M. R. , Liu, S. , Leask, A. , & Seguin, C. A. (2013). Impaired intervertebral disc development and premature disc degeneration in mice with notochord‐specific deletion of CCN2. Arthritis and Rheumatism, 65, 2634–2644. 23839921 10.1002/art.38075

[jcp28952-bib-0007] Bell, S. M. , Zhang, L. , Mendell, A. , Xu, Y. , Haitchi, H. M. , Lessard, J. L. , & Whitsett, J. A. (2011). Kruppel‐like factor 5 is required for formation and differentiation of the bladder urothelium. Developmental Biology, 358, 79–90. 21803035 10.1016/j.ydbio.2011.07.020 PMC3180904

[jcp28952-bib-0008] Bonavita, R. , Vincent, K. , Pinelli, R. , & Dahia, C. L. (2018). Formation of the sacrum requires down‐regulation of sonic hedgehog signaling in the sacral intervertebral discs. Biology Open, 7, bio035592. 29784673 10.1242/bio.035592 PMC6078355

[jcp28952-bib-0009] Bou‐Gharios, G. , Garrett, L. A. , Rossert, J. , Niederreither, K. , Eberspaecher, H. , Smith, C. , … Crombrugghe, B. (1996). A potent far‐upstream enhancer in the mouse pro alpha 2(I) collagen gene regulates expression of reporter genes in transgenic mice. The Journal of Cell Biology, 134, 1333–1344. 8794872 10.1083/jcb.134.5.1333 PMC2120987

[jcp28952-bib-0010] Bowers, M. , Eng, L. , Lao, Z. , Turnbull, R. K. , Bao, X. , Riedel, E. , … Joyner, A. L. (2012). Limb anterior‐posterior polarity integrates activator and repressor functions of GLI2 as well as GLI3. Developmental Biology, 370, 110–124. 22841643 10.1016/j.ydbio.2012.07.017 PMC3432687

[jcp28952-bib-0011] Cantley, L. , Saunders, C. , Guttenberg, M. , Candela, M. E. , Ohta, Y. , Yasuhara, R. , … Zhang, X. (2013). Loss of beta‐catenin induces multifocal periosteal chondroma‐like masses in mice. The American Journal of Pathology, 182, 917–927. 23274133 10.1016/j.ajpath.2012.11.012 PMC3594871

[jcp28952-bib-0012] Chen, M. , Lichtler, A. C. , Sheu, T. J. , Xie, C. , Zhang, X. , O'Keefe, R. J. , & Chen, D. (2007). Generation of a transgenic mouse model with chondrocyte‐specific and tamoxifen‐inducible expression of Cre recombinase. Genesis, 45, 44–50. 17211877 10.1002/dvg.20261 PMC2654410

[jcp28952-bib-0013] Choi, H. , Johnson, Z. I. , & Risbud, M. V. (2015). Understanding nucleus pulposus cell phenotype: A prerequisite for stem cell based therapies to treat intervertebral disc degeneration. Current Stem Cell Research & Therapy, 10, 307–316. 25584906 10.2174/1574888x10666150113112149 PMC4437858

[jcp28952-bib-0014] Choi, K. S. , Cohn, M. J. , & Harfe, B. D. (2008). Identification of nucleus pulposus precursor cells and notochordal remnants in the mouse: Implications for disk degeneration and chordoma formation. Developmental Dynamics, 237, 3953–3958. 19035356 10.1002/dvdy.21805 PMC2646501

[jcp28952-bib-0015] Choi, K. S. , Lee, C. , & Harfe, B. D. (2012). Sonic hedgehog in the notochord is sufficient for patterning of the intervertebral discs. Mechanisms of Development, 129, 255–262. 22841806 10.1016/j.mod.2012.07.003 PMC3478436

[jcp28952-bib-0016] Dahia, C. L. , Mahoney, E. , & Wylie, C. (2012). Shh signaling from the nucleus pulposus is required for the postnatal growth and differentiation of the mouse intervertebral disc. PLOS One, 7, e35944. 22558278 10.1371/journal.pone.0035944 PMC3338762

[jcp28952-bib-0017] Dieleman, J. L. , Baral, R. , Birger, M. , Bui, A. L. , Bulchis, A. , Chapin, A. , … Joseph, J. (2016). US Spending on personal health care and public health, 1996–2013. Journal of the American Medical Association, 316, 2627–2646. 28027366 10.1001/jama.2016.16885 PMC5551483

[jcp28952-bib-0018] Freemont, A. J. (2009). The cellular pathobiology of the degenerate intervertebral disc and discogenic back pain. Rheumatology, 48, 5–10. 18854342 10.1093/rheumatology/ken396

[jcp28952-bib-0019] HALE Collaborators, G. B. D. D. (2015). Global, regional, and national disability‐adjusted life years (DALYs) for 306 diseases and injuries and healthy life expectancy (HALE) for 188 countries, 1990–2013: Quantifying the epidemiological transition. Lancet, 386, 2145–2191. 26321261 10.1016/S0140-6736(15)61340-X PMC4673910

[jcp28952-bib-0020] Harfe, B. D. , Scherz, P. J. , Nissim, S. , Tian, H. , McMahon, A. P. , & Tabin, C. J. (2004). Evidence for an expansion‐based temporal Shh gradient in specifying vertebrate digit identities. Cell, 118, 517–528. 15315763 10.1016/j.cell.2004.07.024

[jcp28952-bib-0021] Hartvigsen, J. , Hancock, M. J. , Kongsted, A. , Louw, Q. , Ferreira, M. L. , Genevay, S. , … Sieper, J. (2018). What low back pain is and why we need to pay attention. Lancet, 391, 2356–2367. 29573870 10.1016/S0140-6736(18)30480-X

[jcp28952-bib-0022] Henry, S. P. , Jang, C. W. , Deng, J. M. , Zhang, Z. , Behringer, R. R. , & de Crombrugghe, B. (2009). Generation of aggrecan‐CreERT2 knockin mice for inducible Cre activity in adult cartilage. Genesis, 47, 805–814. 19830818 10.1002/dvg.20564 PMC3951921

[jcp28952-bib-0023] Henry, S. P. , Liang, S. , Akdemir, K. C. , & de Crombrugghe, B. (2012). The postnatal role of Sox9 in cartilage. Journal of Bone and Mineral Research: The Official Journal of the American Society for Bone and Mineral Research, 27, 2511–2525. 10.1002/jbmr.1696 PMC3502666 22777888

[jcp28952-bib-0024] Hirai, T. , Chagin, A. S. , Kobayashi, T. , Mackem, S. , & Kronenberg, H. M. (2011). Parathyroid hormone/parathyroid hormone‐related protein receptor signaling is required for maintenance of the growth plate in postnatal life. Proceedings of the National Academy of Sciences of the United States of America, 108, 191–196. 21173257 10.1073/pnas.1005011108 PMC3017151

[jcp28952-bib-0025] Hoy, D. , Bain, C. , Williams, G. , March, L. , Brooks, P. , Blyth, F. , … Buchbinder, R. (2012). A systematic review of the global prevalence of low back pain. Arthritis and Rheumatism, 64, 2028–2037. 22231424 10.1002/art.34347

[jcp28952-bib-0026] Kadzik, R. S. , Cohen, E. D. , Morley, M. P. , Stewart, K. M. , Lu, M. M. , & Morrisey, E. E. (2014). Wnt ligand/Frizzled 2 receptor signaling regulates tube shape and branch‐point formation in the lung through control of epithelial cell shape. Proceedings of the National Academy of Sciences of the United States of America, 111, 12444–12449. 25114215 10.1073/pnas.1406639111 PMC4151720

[jcp28952-bib-0027] Kaucka, M. , Petersen, J. , Tesarova, M. , Szarowska, B. , Kastriti, M. E. , Xie, M. , … Symmons, O. (2018). Signals from the brain and olfactory epithelium control shaping of the mammalian nasal capsule cartilage. eLife, 7, 7. 10.7554/eLife.34465 PMC6019068 29897331

[jcp28952-bib-0028] Kishimoto, K. , Tamura, M. , Nishita, M. , Minami, Y. , Yamaoka, A. , Abe, T. , … Morimoto, M. (2018). Synchronized mesenchymal cell polarization and differentiation shape the formation of the murine trachea and esophagus. Nature Communications, 9, 2816. 10.1038/s41467-018-05189-2 PMC6053463 30026494

[jcp28952-bib-0029] Lapinski, P. E. , Bauler, T. J. , Brown, E. J. , Hughes, E. D. , Saunders, T. L. , & King, P. D. (2007). Generation of mice with a conditional allele of the p120 Ras GTPase‐activating protein. Genesis, 45, 762–767. 18064675 10.1002/dvg.20354

[jcp28952-bib-0030] Lapinski, P. E. , Kwon, S. , Lubeck, B. A. , Wilkinson, J. E. , Srinivasan, R. S. , Sevick‐Muraca, E. , & King, P. D. (2012). RASA1 maintains the lymphatic vasculature in a quiescent functional state in mice. Journal of Clinical Investigation, 122, 733–747. 22232212 10.1172/JCI46116 PMC3266774

[jcp28952-bib-0031] Lee, C. R. , Sakai, D. , Nakai, T. , Toyama, K. , Mochida, J. , Alini, M. , & Grad, S. (2007). A phenotypic comparison of intervertebral disc and articular cartilage cells in the rat. European Spine Journal: Official Publication of the European Spine Society, the European Spinal Deformity Society, and the European Section of the Cervical Spine Research Society, 16, 2174–2185. 10.1007/s00586-007-0475-y PMC2140128 17786487

[jcp28952-bib-0032] Levy, V. , Lindon, C. , Harfe, B. D. , & Morgan, B. A. (2005). Distinct stem cell populations regenerate the follicle and interfollicular epidermis. Developmental Cell, 9, 855–861. 16326396 10.1016/j.devcel.2005.11.003

[jcp28952-bib-0033] Liao, L. , Jiang, H. , Fan, Y. , Lu, R. S. , Wei, C. , Takarada, T. , … Chen, D. (2019). Runx2 is required for postnatal intervertebral disc tissue growth and development. Journal of Cellular Physiology, 234, 6679–6687. 30341902 10.1002/jcp.27410 PMC6460473

[jcp28952-bib-0034] Madisen, L. , Zwingman, T. A. , Sunkin, S. M. , Oh, S. W. , Zariwala, H. A. , Gu, H. , … Jones, A. R. (2010). A robust and high‐throughput Cre reporting and characterization system for the whole mouse brain. Nature Neuroscience, 13, 133–140. 20023653 10.1038/nn.2467 PMC2840225

[jcp28952-bib-0035] Maeda, Y. , Nakamura, E. , Nguyen, M. T. , Suva, L. J. , Swain, F. L. , Razzaque, M. S. , … Lanske, B. (2007). Indian hedgehog produced by postnatal chondrocytes is essential for maintaining a growth plate and trabecular bone. Proceedings of the National Academy of Sciences of the United States of America, 104, 6382–6387. 17409191 10.1073/pnas.0608449104 PMC1851055

[jcp28952-bib-0036] Mao, J. , Kim, B. M. , Rajurkar, M. , Shivdasani, R. A. , & McMahon, A. P. (2010). Hedgehog signaling controls mesenchymal growth in the developing mammalian digestive tract. Development, 137, 1721–1729. 20430747 10.1242/dev.044586 PMC2860252

[jcp28952-bib-0037] McCann, M. R. , Tamplin, O. J. , Rossant, J. , & Seguin, C. A. (2012). Tracing notochord‐derived cells using a Noto‐cre mouse: Implications for intervertebral disc development. Disease models & mechanisms, 5, 73–82. 22028328 10.1242/dmm.008128 PMC3255545

[jcp28952-bib-0038] Means, A. L. , Xu, Y. , Zhao, A. , Ray, K. C. , & Gu, G. (2008). A CK19(CreERT) knockin mouse line allows for conditional DNA recombination in epithelial cells in multiple endodermal organs. Genesis, 46, 318–323. 18543299 10.1002/dvg.20397 PMC3735352

[jcp28952-bib-0039] Merceron, C. , Mangiavini, L. , Robling, A. , Wilson, T. L. , Giaccia, A. J. , Shapiro, I. M. , … Risbud, M. V. (2014). Loss of HIF‐1α in the notochord results in cell death and complete disappearance of the nucleus pulposus. PLOS One, 9, e110768. 25338007 10.1371/journal.pone.0110768 PMC4206488

[jcp28952-bib-0040] Minogue, B. M. , Richardson, S. M. , Zeef, L. A. , Freemont, A. J. , & Hoyland, J. A. (2010). Transcriptional profiling of bovine intervertebral disc cells: Implications for identification of normal and degenerate human intervertebral disc cell phenotypes. Arthritis Research & Therapy, 12, R22. 20149220 10.1186/ar2929 PMC2875656

[jcp28952-bib-0041] Mohanty, S. , & Dahia, C. L. (2019). Defects in intervertebral disc and spine during development, degeneration, and pain: New research directions for disc regeneration and therapy. *Wiley Interdisciplinary Reviews. Developmental Biology*, e343. Advance online publication. 10.1002/wdev.343. 10.1002/wdev.343 PMC6565447 30977275

[jcp28952-bib-0042] Munir, S. , Rade, M. , Maatta, J. H. , Freidin, M. B. , & Williams, F. M. K. (2018). Intervertebral disc biology: Genetic basis of disc degeneration. Current Molecular Biology Reports, 4, 143–150. 30464887 10.1007/s40610-018-0101-2 PMC6223888

[jcp28952-bib-0043] Muzumdar, M. D. , Tasic, B. , Miyamichi, K. , Li, L. , & Luo, L. (2007). A global double‐fluorescent Cre reporter mouse. Genesis, 45, 593–605. 17868096 10.1002/dvg.20335

[jcp28952-bib-0044] Mwale, F. (2013). Molecular therapy for disk degeneration and pain. Global Spine Journal, 3, 185–192. 24436869 10.1055/s-0033-1349400 PMC3854687

[jcp28952-bib-0045] Nagao, M. , Cheong, C. W. , & Olsen, B. R. (2016). Col2‐Cre and tamoxifen‐inducible Col2‐CreER target different cell populations in the knee joint. Osteoarthritis and Cartilage, 24, 188–191. 26256767 10.1016/j.joca.2015.07.025 PMC4695246

[jcp28952-bib-0046] Nakamura, E. , Nguyen, M. T. , & Mackem, S. (2006). Kinetics of tamoxifen‐regulated Cre activity in mice using a cartilage‐specific CreER(T) to assay temporal activity windows along the proximodistal limb skeleton. Developmental Dynamics, 235, 2603–2612. 16894608 10.1002/dvdy.20892

[jcp28952-bib-0047] Novais, E. J. , Diekman, B. O. , Shapiro, I. M. , & Risbud, M. V. (2019). p16Ink4a deletion in cells of the intervertebral disc affects their matrix homeostasis and senescence associated secretory phenotype without altering onset of senescence. *Matrix Biology: Journal of the International Society for Matrix Biology*, p16. Advance online publication. 10.1016/j.matbio.2019.02.004. 10.1016/j.matbio.2019.02.004 PMC6708504 30811968

[jcp28952-bib-0048] Ono, N. , Ono, W. , Nagasawa, T. , & Kronenberg, H. M. (2014). A subset of chondrogenic cells provides early mesenchymal progenitors in growing bones. Nature Cell Biology, 16, 1157–1167. 25419849 10.1038/ncb3067 PMC4250334

[jcp28952-bib-0049] Park, E. J. , Sun, X. , Nichol, P. , Saijoh, Y. , Martin, J. F. , & Moon, A. M. (2008). System for tamoxifen‐inducible expression of cre‐recombinase from the Foxa2 locus in mice. Developmental Dynamics, 237, 447–453. 18161057 10.1002/dvdy.21415

[jcp28952-bib-0050] Risbud, M. V. , Schoepflin, Z. R. , Mwale, F. , Kandel, R. A. , Grad, S. , Iatridis, J. C. , … Hoyland, J. A. (2015). Defining the phenotype of young healthy nucleus pulposus cells: Recommendations of the spine research interest group at the 2014 annual ORS meeting. Journal of Orthopaedic Research: Official Publication of the Orthopaedic Research Society, 33, 283–293. 25411088 10.1002/jor.22789 PMC4399824

[jcp28952-bib-0051] Sohn, P. , Cox, M. , Chen, D. , & Serra, R. (2010). Molecular profiling of the developing mouse axial skeleton: A role for Tgfbr2 in the development of the intervertebral disc. BMC Developmental Biology, 10, 29. 20214815 10.1186/1471-213X-10-29 PMC2848151

[jcp28952-bib-0052] Sugimoto, Y. , Takimoto, A. , Hiraki, Y. , & Shukunami, C. (2013). Generation and characterization of ScxCre transgenic mice. Genesis, 51, 275–283. 23349075 10.1002/dvg.22372

[jcp28952-bib-0053] Szymaniak, A. D. , Mi, R. , McCarthy, S. E. , Gower, A. C. , Reynolds, T. L. , Mingueneau, M. , … Varelas, X. (2017). The Hippo pathway effector YAP is an essential regulator of ductal progenitor patterning in the mouse submandibular gland. eLife, 6, 6. 10.7554/eLife.23499 PMC5466420 28492365

[jcp28952-bib-0054] Uetzmann, L. , Burtscher, I. , & Lickert, H. (2008). A mouse line expressing Foxa2‐driven Cre recombinase in node, notochord, floorplate, and endoderm. Genesis, 46, 515–522. 18798232 10.1002/dvg.20410

[jcp28952-bib-0055] Urban, J. P. , Holm, S. , & Maroudas, A. (1978). Diffusion of small solutes into the intervertebral disc: As in vivo study. Biorheology, 15, 203–221. 737323 10.3233/bir-1978-153-409

[jcp28952-bib-0056] Urban, J. P. , & Roberts, S. (2003). Degeneration of the intervertebral disc. Arthritis Research & Therapy, 5, 120–130. 12723977 10.1186/ar629 PMC165040

[jcp28952-bib-0057] Urban, J. P. , & Winlove, C. P. (2007). Pathophysiology of the intervertebral disc and the challenges for MRI. Journal of Magnetic Resonance Imaging, 25, 419–432. 17260404 10.1002/jmri.20874

[jcp28952-bib-0058] Vincent, K. , Mohanty, S. , Pinelli, R. , Bonavita, R. , Pricop, P. , Albert, T. J. , & Dahia, C. L. (2019). Aging of mouse intervertebral disc and association with back pain. Bone, 123, 246–259. 10.1016/j.bone.2019.03.037 30936040 PMC6549718

[jcp28952-bib-0059] Weiler, C. , Nerlich, A. G. , Schaaf, R. , Bachmeier, B. E. , Wuertz, K. , & Boos, N. (2010). Immunohistochemical identification of notochordal markers in cells in the aging human lumbar intervertebral disc. European spine journal: Official Publication of the European Spine Society, the European Spinal Deformity Society, and the European Section of the Cervical Spine Research Society, 19, 1761–1770. 10.1007/s00586-010-1392-z PMC2989227 20372940

[jcp28952-bib-0060] Whitfield, J. , Littlewood, T. , & Soucek, L. (2015). Tamoxifen administration to mice. Cold Spring Harbor protocols, 2015, 269–271. 25734062 10.1101/pdb.prot077966 PMC6773604

[jcp28952-bib-0061] Winkler, T. , Mahoney, E. J. , Sinner, D. , Wylie, C. C. , & Dahia, C. L. (2014). Wnt signaling activates Shh signaling in early postnatal intervertebral discs, and re‐activates Shh signaling in old discs in the mouse. PLOS One, 9, e98444. 24892825 10.1371/journal.pone.0098444 PMC4043533

[jcp28952-bib-0062] Wong, J. , Sampson, S. L. , Bell‐Briones, H. , Ouyang, A. , Lazar, A. A. , Lotz, J. C. , & Fields, A. J. (2019). Nutrient supply and nucleus pulposus cell function: Effects of the transport properties of the cartilage endplate and potential implications for intradiscal biologic therapy. Osteoarthritis and Cartilage, 27, 956–964. 30721733 10.1016/j.joca.2019.01.013 PMC6536352

[jcp28952-bib-0063] Wu, W. J. , Zhang, X. K. , Zheng, X. F. , Yang, Y. H. , Jiang, S. D. , & Jiang, L. S. (2013). SHH‐dependent knockout of HIF‐1 α accelerates the degenerative process in mouse intervertebral disc. International Journal of Immunopathology and Pharmacology, 26, 601–609. 24067457 10.1177/039463201302600304

[jcp28952-bib-0064] Zheng, Y. , Fu, X. , Liu, Q. , Guan, S. , Liu, C. , Xiu, C. , … Zhang, Z. (2019). Characterization of Cre recombinase mouse lines enabling cell type‐specific targeting of postnatal intervertebral discs. Journal of Cellular Physiology, 234, 14422–14431. 10.1002/jcp.28166 PMC6650379 30675722

[jcp28952-bib-0065] Zheng, Y. , Liu, C. , Ni, L. , Liu, Z. , Mirando, A. J. , Lin, J. , … Li, B. (2018). Cell type‐specific effects of Notch signaling activation on intervertebral discs: Implications for intervertebral disc degeneration. Journal of Cellular Physiology, 233, 5431–5440. 30025440 10.1002/jcp.26385 PMC6092956

[jcp28952-bib-0066] Zhou, X. , von der Mark, K. , Henry, S. , Norton, W. , Adams, H. , & de Crombrugghe, B. (2014). Chondrocytes transdifferentiate into osteoblasts in endochondral bone during development, postnatal growth and fracture healing in mice. PLOS Genetics, 10, e1004820. 25474590 10.1371/journal.pgen.1004820 PMC4256265

[jcp28952-bib-0067] Zito, G. , Saotome, I. , Liu, Z. , Ferro, E. G. , Sun, T. Y. , Nguyen, D. X. , … Greco, V. (2014). Spontaneous tumour regression in keratoacanthomas is driven by Wnt/retinoic acid signalling cross‐talk. Nature Communications, 5, 3543. 10.1038/ncomms4543 PMC3974217 24667544

